# Variants of uncertain significance in *BRCA*: a harbinger of ethical and policy issues to come?

**DOI:** 10.1186/s13073-014-0121-3

**Published:** 2014-12-19

**Authors:** Jae Yeon Cheon, Jessica Mozersky, Robert Cook-Deegan

**Affiliations:** Center for Public Genomics, Duke University, Box 90141, 304 Research Drive, Durham, NC 27708-0141 USA; The New School for Social Research, 6 East 16th Street, Office 921, New York, NY 1003 USA; Center for the Integration of Genetic Healthcare Technologies (CIGHT), University of Pennsylvania, Philadelphia, PA 19104 USA

## Abstract

After two decades of genetic testing and research, the *BRCA1* and *BRCA2* genes are two of the most well-characterized genes in the human genome. As a result, variants of uncertain significance (VUS; also called variants of unknown significance) are reported less frequently than for genes that have been less thoroughly studied. However, VUS continue to be uncovered, even for *BRCA1*/*2*. The increasing use of multi-gene panels and whole-genome and whole-exome sequencing will lead to higher rates of VUS detection because more genes are being tested, and most genomic loci have been far less intensively characterized than *BRCA1*/*2*. In this article, we draw attention to ethical and policy-related issues that will emerge. Experience garnered from *BRCA1*/*2* testing is a useful introduction to the challenges of detecting VUS in other genetic testing contexts, while features unique to *BRCA1*/*2* suggest key differences between the *BRCA* experience and the current challenges of multi-gene panels in clinical care. We propose lines of research and policy development, emphasizing the importance of pooling data into a centralized open-access database for the storage of gene variants to improve VUS interpretation. In addition, establishing ethical norms and regulated practices for sharing and curating data, analytical algorithms, interpretive frameworks and patient re-contact are important policy areas.

**Electronic supplementary material:**

The online version of this article (doi:10.1186/s13073-014-0121-3) contains supplementary material, which is available to authorized users.

## Background

There is an increasing move towards the use of multi-gene panels, whole-genome sequencing (WGS) and whole-exome sequencing (WES) in clinical care. In contrast to genetic testing for mutations in a single gene or in a limited number of high-penetrance genes associated with a particular disease (such as *BRCA1*/*2*), multi-gene panels and WGS involve testing numerous variably penetrant genes or the entire genome. One consequence of using panels or WGS/WES is that genetic testing results are more likely to include variants of uncertain significance (VUS). A key challenge is that the clinical significance of a VUS result for disease risk is by definition unclear. This makes clinical management recommendations more complex, while also potentially creating anxiety or misunderstanding among patients. Further studies of the variant in question may lead to a VUS result being reclassified as either deleterious or not, raising policy and ethical questions about the duty to re-contact patients and who is ultimately responsible for this task. The methods of VUS classification and reclassification vary and are the subject of multiple international efforts, although there remain no universally accepted standards or methods for determining pathogenicity and reporting VUS results [1]. Collaborative efforts to collate data on VUS are essential to improving the current difficulties created by VUS results.

*BRCA1*/*2* genetic testing, which has been in use for nearly two decades, still produces VUS results regularly [2]. Multi-gene panels and WGS/WES will inevitably increase the number of VUS found because more genes are included in the analyses [3,4]. In comparison with *BRCA1*/*2*, there will be far less information regarding new VUS results because far fewer genetic tests and studies have been completed for other genes.

The experience garnered from *BRCA* gene testing can help inform how new developments in genomics will play out, not only for *BRCA* genes, but for others as well. At the same time, specific unique features of *BRCA1*/*2* - such as the longstanding patent held by Myriad Genetics in the United States until recently, as well as the great length and short exon sequences of these two genes - also suggest key differences between the *BRCA* experience and the current challenges of multi-gene panels and WGS in clinical care. Interpreting the clinical meaning of newly discovered variants will be one of the major challenges of ‘genomic’, or ‘precision’, medicine.

In this article, we identify some of the clinical, laboratory and data management difficulties that are presented by VUS found during *BRCA* gene testing, and draw attention to ethical, legal and policy-related issues that will emerge as more testing is carried out on an increasing number of genes. We then suggest key areas for research and policy development, particularly the importance of a centralized open-access database for the storage of gene variants.

## Experiences from *BRCA*: VUS results and challenges for clinical management

The *BRCA1* and *BRCA2* genes were initially sequenced and characterized two decades ago, in 1994 and 1995, respectively [5-8]. They have since become two of the most thoroughly studied genes in the human genome. Deleterious mutation carriers have a significantly increased lifetime risk of breast and ovarian cancer, as well as other cancers [9]. Genetic testing to determine whether an individual harbors a deleterious *BRCA1*/*2* mutation plays an important role in assessing risk and determining clinical management [10,11].

However, even for well-studied genes like *BRCA1*/*2*, new VUS continue to appear. A VUS result means that although the testing laboratory detected a DNA alteration, there was not enough evidence to classify that alteration as deleterious or neutral. In the case of *BRCA*, a VUS result gives no clear indication as to whether or not the patient is at higher risk for developing breast or ovarian or another cancer, and further studies are necessary to determine the significance (or not) of the variant in question.

International collaborative studies of the clinical meaning and significance of VUS results in the context of *BRCA1*/*2* testing, as well as the ethical and policy implications, have been ongoing since the 1990s [1,12-15]. These include efforts by the Breast Cancer Information Core (BIC) Working Group (part of the US National Human Genome Research Institute (NHGRI)) and the International Agency for Research on Cancer (IARC) Unclassified Genetic Variants Working Group (part of the World Health Organization)[16-18].

In the United States, Myriad Genetics was the only commercial testing laboratory for *BRCA1*/*2* until the June 2013 Supreme Court ruling that overturned the patentability of genomic DNA [19]. This enabled Myriad to collect the largest single proprietary database of *BRCA1*/*2* testing results, and one consequence was a low VUS rate: in 2013, Myriad reported a VUS rate of 2.1% [2]. Immediately after the Supreme Court ruling, several laboratories began offering *BRCA* testing or announced that they would do so. VUS rates for *BRCA1*/*2* tests and VUS reclassification procedures vary among the different gene testing laboratories now offering testing (Additional file 1) [20]. For instance, Ambry Genetics reports a VUS rate of 3.64% [21], while some European laboratories have reported a 15% VUS rate for *BRCA1*/*2* [22]. Certain for-profit companies, such as Myriad, consider the databases, algorithms and processes through which variant classification occurs to be proprietary information. As a result, specific details are often not released, and VUS rates cannot be independently verified. However, several general strategies for classifying variants and making informed predictions about disease risk are known [2,14,15,22,23]. International academic collaborations of clinicians and scientists, such as the ENIGMA consortium, are working to define a rigorous and consistent approach for variant classification [15].

Various lessons can be gleaned from the *BRCA1*/*2* experience, especially with regard to Myriad Genetics. Over time, Myriad’s large centralized database enabled variants to be initially classified as VUS and then efficiently reclassified as being more likely to be deleterious or neutral - a testament to the utility of a large, well-curated, centralized database [2]. However, the proprietary nature of Myriad’s database meant that it was inaccessible to many researchers and clinicians, and testing by other laboratories who lack access to the database continues to produce higher VUS rates - a testament to the importance of data sharing and collation by all testing providers, and an example of the challenges created by proprietary databases [24].

The classification and reporting of genetic testing results vary internationally (Table 1). The IARC Working Group suggests a five category system for reporting sequence variations: pathogenic, likely pathogenic, uncertain, likely neutral, and neutral [18]. Similarly, the Dutch and British societies for clinical molecular genetics suggest reporting sequence variants in five classes: (1) clearly not pathogenic; (2) unlikely to be pathogenic; (3) unknown significance (VUS); (4) likely to be pathogenic; and (5) clearly pathogenic [25]. The American College of Medical Genetics (ACMG) proposes six categories: (1) previously reported and causative of the disorder; (2) not previously reported but expected to cause the disorder; (3) not previously reported and may or may not cause the disorder; (4) not previously reported but probably does not cause the disorder; (5) previously reported and known to be neutral; and (6) not expected to cause the disorder but reported to be associated with a clinical presentation [26]. The ACMG category (1) and the IARC ‘pathogenic’ classification both refer to a deleterious mutation, a genetic variation or sequence alteration that causes an elevated risk of disease. A negative or neutral test result does not necessarily indicate that there is no increased risk, especially in the context of a compelling family history. Rather, it indicates that elevated risk cannot be attributed to known mutations in *BRCA1*/*2* genes. However, a negative test result can still be informative when a patient has tested negative for a particular deleterious mutation already known to run in his/her family. The ACMG category (3) and the IARC ‘uncertain’ classification both indicate a VUS result, meaning that while the analysis detected a variation in the sequence, the causal relationship between this particular variation and hereditary risk of disease is unclear (Table 1).

**Table 1:** **Examples of recommendations for genetic variant classification**

**Working group affiliation**	**National affiliations of participating researchers**	**Dates of approval, publishing, or update**	**Proposed number of variant classifications**	**Proposed classification categories**
American College of Medical Genetics [26]	United States	2007; revisions published April 2008	6	(1) Previously reported and is causative of the disorder
				(2) Not previously reported but expected to cause the disorder
				(3) Not previously reported and may or may not cause the disorder
				(4) Not previously reported but probably does not cause the disorder
				(5) Previously reported and known to be neutral
				(6) Not expected to cause the disorder but reported to be associated with a clinical presentation^a^
European Society of Human Genetics [35]	France, Czech Republic, Belgium, Switzerland, the Netherlands, Germany, Slovenia, Italy, United Kingdom, Ireland	Published online August 2013; published in the *European Journal of Human Genetics* 2014	5	Normal (physiological finding, normal variation) findings
				Non-specific findings without clinical relevance
				Incidental findings with possible clinical relevance
				Findings of uncertain significance
				Pathognomonic (disease-specific, pathological) findings
International Agency for Research on Cancer [18] (World Health Organization)	Australia, Canada, France, Italy, the Netherlands, United Kingdom, United States	November 2008	5	Pathogenic
				Likely pathogenic
				Uncertain
				Likely neutral
				Neutral
UK Clinical Molecular Genetics Society and Association of Clinical Cytogenetics [25]	United Kingdom	Ratified 11 January, 2008; update approved and published September 2013	5	(1) Clearly not pathogenic
				(2) Unlikely to be pathogenic
				(3) Unknown significance (VUS)
				(4) Likely to be pathogenic
				(5) Clearly pathogenic
Dutch Society of Clinical Genetic Laboratory Specialists [25]	The Netherlands	Ratified 22 October, 2007; update approved and published September 2013		

^a^Categories have been summarized for brevity. VUS, variants of uncertain significance.

A true VUS, with insufficient evidence to clearly indicate pathogenicity or neutrality, is treated as clinically uninformative. In this case, other information, such as family history, is used to make clinical management decisions [11,27,28]. In practice, ACMG categories (2) and (3) are sometimes combined or managed similarly because neither is considered to be a clinically actionable result. However, there has been some controversy over the clinical management of a result that is ‘likely pathogenic’ or ‘expected to cause the disorder’ and whether such results should essentially be treated as a VUS or as a deleterious result [14,18,26,29].

## Methods of VUS reclassification

VUS results can subsequently be reclassified as likely deleterious or likely neutral as more information becomes available. Reclassification procedures and methods vary among laboratories and can include the following: assembling evidence from additional testing of relatives, discovery of the same variants in other families, biological methods such as RNA transcript analysis, creating knock-in or knock-out animal models, or performing gene rescue studies. Another method is finding variants in tandem with known deleterious mutations, because two deleterious mutations are generally incompatible with surviving past embryonic development [30]. Genetic, epidemiological and histopathological features, and animal model, *in vitro* (tissue culture) and *in silico* (bioinformatic) analyses may all be used to reclassify variants [31]. Finding variants in large numbers of people who are unaffected, as reflected in a high allele frequency in population studies, for example, is a powerful method for reclassifying variants as unlikely to be deleterious [32]. However, the large number of individuals required for such studies will be a challenge when studying variants that are very rare in the population [31]. Various probability and multi-factorial models have also been proposed for the reclassification of variants [1,14,22,33]. These and other considerations help classify an initial VUS as likely to be a deleterious mutation or likely to be a neutral polymorphism.

Guidelines for the classification of genetic variants have been proposed for both research and clinical purposes. Although there is no consensus regarding procedures for the reclassification of VUS, the main guidelines used in the United States are the recommendations and updates from the ACMG [26,34], while analogous European guidelines have been proposed by the European Society of Human Genetics [35]. In 2008, the ACMG issued a revision of the 2007 standards for reporting sequence variations, which specified interpretive categories for sequence variations to be used in clinical reports [26]. Categorization is based on several factors, such as whether the variation causes a nonsense, missense or frameshift mutation in the genetic coding sequence. Other factors are the location of the variation in the exon or intron sequence and the possibility that the change in sequence could produce a new splice site or eliminate a known splice site. However, large-scale chromosomal rearrangements, such as inversions, deletions and insertions, may be missed by some methods, and their effect on gene function can be difficult to predict.

## The logic of testing multiple genes despite high rates of VUS

Two decades of *BRCA* testing and research have produced *BRCA* VUS rates that are lower than for most other genes. The move to testing many genes in a ‘panel’ (also known as multi-gene or multiplex testing), or performing WGS/WES, increases the likelihood that a VUS will be detected in at least one gene because far more genes are being tested, and variants uncovered in other genes are more likely never to have been seen [3,4,36].

Many laboratories, both commercial (such as Myriad Genetics, Ambry Genetics, GeneDx, Invitae, Quest, LabCorp, Counsyl and others [37-43]) and academic (such as those affiliated with the University of Washington, University of California Los Angeles, University of Chicago, Emory University and others [44-47]), are now offering multi-gene testing, WES and/or WGS. For some examples of *BRCA-*related multi-gene testing, see Additional file 1. These tests allow for simultaneous analysis of multiple genes (multiplex testing) or the entire genome at a cost that is often only slightly greater than a single gene test [20,48]. These tests may involve sequencing various numbers of genes, from 2 to over 100 or 200 genes, the entire exome (selecting for protein-coding sequences) or the entire genome [48]. Panels and WGS analyses are being used as predictive and diagnostic tests in oncology, cardiology and other clinical contexts.

Multi-gene testing can lead to additional findings of clinically relevant information. There have been cases in which multi-gene panels detected pathogenic mutations that would not have been identified if *BRCA1*/*2* testing alone had been used, leading to a change in care and the early detection of cancers [4]. The use of WGS and WES seems particularly promising in the treatment of patients who have been on diagnostic odysseys and are suspected to suffer from rare Mendelian (single-gene) conditions [49]. While WGS and multi-gene panels have the potential to increase clinically relevant findings, such testing substantially increases the volume of detected variants per individual, and the interpretation of these requires significant clinical and laboratory labor [3,36,50].

Despite the difficulties, the logic of testing multiple genes remains compelling. Cancer is a complex genetic disease. Breast and ovarian cancers, for example, are associated with many genes, not just *BRCA1*/*2* [11]. Other cancers, apart from breast and ovarian cancer, are also associated with *BRCA1*/*2* mutations [51,52]. The pleiotropy of cancer-associated mutations raises questions about penetrance, gene interactions and other factors, all of which complicate clinical interpretation. With the power of sequence analysis increasing and the cost plummeting, multi-gene panels, WES and WGS are now used in both research and clinical care [49,53,54]. This is especially prevalent in fields already using genomics in the clinical setting, particularly in the treatment of cancer, cardiac disease and diabetes. With sequencing costs continuing to drop, current tests that examine single genes or just a few genes may eventually be replaced by panels, WES, WGS, or other methods that provide far more information for similar or lesser cost.

We are not advocating for immediate and indiscriminate multi-gene testing without regard to patient history and other relevant context. However, gene panels are already on the market, and it is necessary to consider how to make the best of the current situation, in which sequencing capacity has outpaced interpretive ability.

## Ethical issues and policy implications

VUS results raise numerous ethical and policy issues (Table 2). In particular, the uncertainty raised by VUS results may lead to misunderstanding by patients and professionals, while the issue of where responsibility lies regarding the duty to re-contact patients if new information arises remains unresolved. In the context of *BRCA1*/*2* testing, it is unclear how patients understand and deal with the uncertainty raised by a VUS result [55,56]. Do they understand what VUS results do - or do not - mean? How do different patients react? Some reports suggest that patients may misinterpret a VUS result as non-deleterious, while clinicians may misinterpret them as deleterious and make potentially inappropriate management suggestions [57]. Furthermore, what do clinicians and genetic counselors do to follow up VUS results? Do they apply bioinformatic interpretation tools? Do they submit variants for further analysis using biological methods or in animal models? Do they notify members of the ENIGMA consortium [15]? What do healthcare providers discuss with their current patients and how could we help them to better serve patients? Policies to encourage data sharing and curation can help to speed up the rate of VUS interpretation, and avoid some of the pitfalls of proprietary databases, as exemplified by the Myriad experience.

**Table 2:** **Major policy issues**

**ELSI challenges**	**Policy questions**	**Possible solutions**
Duty to re-contact	To what extent healthcare providers have a duty to re-contact patients in the case of a reclassified VUS	• EHRs and integration of genomic data into EHRs
		• Rely on patient to request new interpretation
		• Patient access to databases
		• Formulate standard practices for re-contact
		• Integrate notification of users (health professionals, counselors or consumers) into databases, websites and interpretive software
Informed consent	Considerations and options within the informed consent process	• Broad consent
		• Opt out option for some results
		• Options for re-contact integrated into consent process
Patient understanding	How to reduce uncertainty introduced by VUS results that can lead to misunderstanding by patients and professionals	• Careful pretest counseling with qualified genetic counselors or other health professionals
		• Education of clinicians, counselors and consumers
		• Resources to assist consumers in interpreting test results
Data sharing	How to aggregate data on VUS from disparate clinical and research laboratories, particularly those performing gene panel or WES/WGS analyses	• Centralized open-access database
		• Make deposition of data and methods sufficient to enable independent verification a condition of payment
		• Accreditation of laboratories contingent on independent verification and data sharing
		• Condition of certification for laboratory directors and/or genetics health professionals
Research to improve VUS interpretation	How to improve the evidence base for interpretation of genomic variants	• Public and private research programs
		• Individual research projects
		• Consortia and formal research networks
		• Studies of ethical, legal and practical experience in using gene panels and WGS/WES analysis

EHR, electronic health record; ELSI, ethical, legal and social implication; VUS, variants of uncertain significance; WES, whole-exome sequencing; WGS, whole-genome sequencing.

## Duty to re-contact

A lack of consensus about when and whether a physician, researcher or genetic counselor has a duty to re-contact patients upon variant reclassification adds an additional layer of ethical and logistical complexity to the technical uncertainty [58]. When new information that sheds light on the clinical significance of a variant is discovered, is it the responsibility of the laboratory, the clinician or the genetic counselor to contact the person who carries the mutation? Or is it the responsibility of the affected person to periodically check back? Where does responsibility lie, and what degree of evidence triggers a need to share newfound information?

The problem of re-contacting patients is far from simple. At the least, genetic testing generally involves a clinician, a patient (or an entire family), and a testing laboratory. Any one of these may change address or may no longer be performing the same duties when a VUS interpretation changes. Where should responsibility lie for relaying information about reclassification - with the patient, the laboratory, the genetic counselor, the clinician or some combination?

Currently, some laboratories report VUS reclassifications to the healthcare provider or genetic counselor who ordered the test. It is then that health professional’s responsibility to get back in touch with the patient. A problem with this system arises when healthcare providers have out of date patient contact information, resulting in an inability to re-contact the patient or family. Wasted time and resources at the stage of re-contacting the patient will become more significant as multi-gene panels produce high rates of VUS and subsequent reclassification. Some groups may not have the manpower or resources needed to follow up reclassified VUS results for all tested patients. In addition, not all laboratories have systems in place for ensuring that reclassified variants are regularly reported back to the healthcare professional who ordered the test (Additional file 1) [20]. Current practices are incorporating the capacity to relay new information about variant calls as it becomes available, but the process is not uniform or universal.

Additional pragmatic issues arise when a physician or genetic counselor leaves a particular practice, or if a practice closes entirely, making re-contact with the associated patients difficult, if not impossible. Electronic health records (EHRs) have the potential to help, in that medical records, including test results, would be electronically available across sites of care. However, interoperability and compatibility across different platforms remain substantial hurdles for EHR systems, so that results from tests ordered at one site may not be transferred to an alternative EHR system. Because some genetic testing is also done via smaller individual clinics which may use different, or not have any, EHR systems, as opposed to comparatively larger healthcare organizations, these issues could significantly contribute to long-term inconsistencies in handling patient medical records and maintaining patient contact. The practices and infrastructure to enable reclassification are under construction, as are those necessary for conveying reclassification information to patients getting testing and the professionals who provide their health care.

In the context of multi-gene panels and WES/WGS, issues regarding the scale and complexity of VUS reclassification are compounded and magnified [36]. This is because the other genes on these panels are far less studied than *BRCA1*/*2* and, upon testing, are much more likely to produce VUS results. While some may argue that this is a reason not to do gene panels until further research is completed, the fact remains that multi-gene panels are already being introduced into clinical use by many laboratories, and they are being demanded by healthcare providers and patients alike [48,59]. Indeed, the management of VUS results will be one of the core features of a multi-institutional Prospective Registry of Multiplex Testing (PROMPT) study at the Mayo Clinic, Memorial Sloan Kettering Cancer Center, and the University of Pennsylvania [60].

## Data-sharing and curation of databases

Commercial, academic and non-profit laboratories are using the availability of additional genes in a single panel as a marketing tool - a ‘more is better’ approach. In the United States, the rapid uptake of multi-gene tests may be partly attributable to the highly commercialized nature of medicine and health care, increasing the likelihood of competitive pressure resulting in tests being introduced into clinical care before there is a well-established evidence base for optimal use [53,61-63]. While the VUS rate should drop over time, the prospect of dealing with many VUS results will confront the system immediately and for the foreseeable future [3,4,36]. Healthcare providers and patients will continue to face uncertainty while making life-changing decisions about treatment. With current and future patients in mind, how can we lower the VUS rates as fast as possible?

The key to improving the interpretation of VUS results, and thus lowering the overall VUS rate, is the accumulation and organization of high-quality data and the development of robust methods for data interpretation. The data will come as a result of testing large numbers of patients and following up on their clinical outcomes. In the case of *BRCA1*/*2*, Myriad’s previous patent position led to the company performing the vast majority of testing in the United States. This allowed Myriad to accumulate and collate genomic, pedigree and clinical data, enabling them to maintain the lowest VUS rate achieved so far. Myriad contributed to the BIC database until 2006 (with their last large data contribution in November 2004), but thereafter maintained a proprietary database, largely inaccessible to academic researchers, non-profit organizations and the patients who had sent their samples for analysis.

The situation for multi-gene panels and WES/WGS will be different because there are many genetic testing companies on the market already offering their own gene panels or sequencing services. Competition among companies has led to more competitive pricing, but also to multiple places in which data are held, some of which may remain proprietary and therefore not widely accessible. We suggest that the fastest way to accumulate data and lower VUS rates is to ensure that all of the data eventually go to one accessible place. How can we encourage this outcome in a competitive marketplace?

A significant part of the solution to this problem is the creation of a centralized database for gene variants. This does not imply that all data of all kinds should be in a single database; rather, we suggest the creation of a database designed for clinical use containing the core data used by those interpreting genomic variants. Reclassification of variants happens using a variety and combination of methods, including functional assays, pedigree/family histories and statistical analyses. The importance of a centralized database is in the convergence of these many pieces of data for a meaningful and accurate reclassification of VUS, although even a large centralized database does not eliminate the need for additional clinical interpretation, statistical analyses and further research. The ClinVar database, an open-access National Center for Biotechnology Information (NCBI)-funded resource connecting genome variations and phenotypes, has the potential to fulfill this role [64], in a similar manner to the BIC and others [31], but with a clinical focus that also includes a greater range of genetic disorders. However, its construction faces a number of ethical, commercial, legal and logistical obstacles. Some of the legal and ethical issues concern the maintenance of patient privacy and ensuring informed consent. ClinVar and the laboratories and research institutions collaborating with and contributing to it are working to address these issues. From an intellectual property perspective, why would entities with monetary or commercial interests be willing to share proprietary information? The main logistical issues concern the lack of existing infrastructure for data sharing, the development of interpretive algorithms, and obtaining funding to maintain the database.

There are currently many groups working to address some of these data-sharing issues, particularly by creating repositories in which data can be collated [15,64-69]. Such efforts have not yet converged on a single centralized database, although as previously mentioned, ClinVar may emerge as the main hub for clinical interpretation. These efforts have also been hindered because not all databases are up to date and reliable, and some locus-specific databases are small operations [31,70]. This is unsurprising given that many of the databases were established to enable research, and funding is unstable or insufficient to support use as a reliable clinical tool. Policies that create incentives for participation in public databases are the most promising way to expedite clinical interpretation of genomic data in the long run.

Competition in the genetic testing market means that insurance companies, health plans and other payers (government or otherwise) have a strong influence. The degree of competition in genetic testing depends on how a healthcare system is organized. *BRCA* testing in the United Kingdom, for example, remained largely under the auspices of the National Health Service, despite Myriad’s UK patent rights [71]. In Canada, Myriad’s effort to enforce its patents through its licensee was resisted by the Health Ministry (and ultimately the Premier) of the Province of Ontario, which refused to force its provincial health system to stop *BRCA* testing. Myriad never sued, so in effect Myriad’s patents in Canada have not been enforced [72]. Children’s Hospital of Eastern Ontario recently filed a lawsuit contesting patents on genes associated with long QT syndrome [73]. One purpose of the litigation is to clarify Canadian law on whether genes can be patented. And in Australia, laboratories under the provincial health systems continue to offer *BRCA* testing under a voluntary agreement by the *BRCA*-testing licensee [74]. A recent review of the effects of patents on genetic testing in Australia paints a nuanced picture and cautions ‘against extrapolating [views on effects of patents on genetic testing] survey results from one jurisdiction to another’ [75]. The role of patents thus differs among jurisdictions, but the central importance of pooling data and sharing methods is global.

Policy change could encourage the sharing of data needed to make and verify clinical interpretations of genetic variants. For instance, a requirement for reimbursement could be that laboratories offering tests must share sufficient information about methods and sufficient data for independent verification of results and interpretation. Such a policy could be an effective method of encouraging laboratories to contribute to public databases and to share their interpretive algorithms. Another policy option is to pay directly for interpretive services, but only on condition of sufficient disclosure to enable independent verification. These policy changes could be implemented either as criteria for accreditation (such as those set by the International Organization for Standardization (ISO) in much of Europe; Clinical Pathology Accreditation (CPA) in the United Kingdom, although CPA accreditation is currently being transitioned over to ISO; laboratory accreditation under the College of American Pathologists), certification of health professionals (such as those set by the Clinical Laboratory Improvement Amendments in the United States), or as a condition of laboratory reimbursement stipulated by insurers and health plans that pay for the tests [12,16,76,77].

How and when to re-contact patients if new information emerges could also become more consistent as a matter of policy. One option is the effective implementation of EHRs, in which a patient could directly update contact information on a single record that stays with him or her, regardless of the specific healthcare provider. This would, however, also require the integration of clinically relevant genomic data into the EHR, which remains a challenge. Even with the infusion of public funding, the implementation of a well-functioning, reliable EHR system requires significant additional investment, and wide-scale implementation has proved to be difficult [62,78-80]. Informed consent procedures that clearly indicate that it is a patient’s responsibility to periodically check back in with their provider after a VUS result would put the onus on patients, and avoid some of the pitfalls of patients changing address or changing physicians. Another option is to design a database that manages genotypes and phenotypes so that interested patients can search for updates themselves. In the event of a reclassification, they could re-contact their healthcare providers to discuss further options. Direct consumer use of genotype databases would, however, present a daunting technical challenge because users would require a simplified interface designed for infrequent and non-expert users, not just for genetic professionals. The Patient-Centered Outcomes Research Institute (PCORI) has announced plans to spend US$100 million building PCORnet to empower individuals and families to participate in research. Several genetic organizations have joined early efforts to harness this power, including PCORnet, the Genetic Alliance, Facing Our Risk of Cancer Empowered and DuchenneConnect [81-84]. These efforts will help pave the way to stronger patient engagement, and will provide lessons on how best to manage patient re-contact, informed consent and access to clinical data and databases.

Another important policy to improve the clinical interpretation of genomic variation is arguably the most important of all: continuing support for research efforts that have been sustained for three decades on developing methods for functional biological analysis, fostering bioinformatic methods, and detecting such variants. These efforts are global, and include networks in Europe, North America and Asia, and increasing attention to ‘translational’ research efforts, in moving from genomic discovery to clinical utility.

## Conclusions

In this article, we use some lessons learned from *BRCA1*/*2* testing to anticipate challenges that will emerge from the rise in VUS results due to the increasing use of multi-gene panels and WES/WGS. Experience with *BRCA1*/*2* is helpful, but not fully generalizable. The *BRCA1*/*2* genes are large and mutations in them are spread widely, and the unusual - perhaps unique - US service monopoly that gave rise to a proprietary database might not presage experience with other genes. But the central point is that standards for informed consent, how and when to inform those who have VUS that their variants have been reclassified, and the infrastructure for storing and interpreting genomic variants are truly important, and policies are required to build the requisite infrastructure and develop the data-sharing and curation practices that will be needed.

While international efforts to classify and reclassify variants are ongoing, the system for reclassifying and handling VUS results in clinical care is evolving rapidly. It is not entirely consistent and some elements, such as data-sharing practices, are fragile and incomplete. Although there will be higher rates of VUS as more unfamiliar genes are tested as part of panels, the logic for testing many genes in an effort to understand complex diseases remains compelling. Ethical, legal and policy implications center on data sharing, curation, patient re-contact and incentives for contributing data to public databases. We argue that an open-access centralized database is a necessary element, although it will not itself be sufficient, in efforts to reclassify VUS in the shortest amount of time possible.

Beyond infrastructure, there is a pressing need for further research on the social, ethical and legal implications of VUS results for patients and healthcare providers who are already dealing with VUS, multiplex panels and WES/WGS on the ground. Such work is underway in many of the large-scale clinical sequencing grant programs in North America and Europe. Uncertainty will persist until many genes in many people have been analyzed and longitudinal studies have been completed on their long-term health outcomes.

Ensuring the rapid and effective sharing of data and methods will require investment to build the necessary infrastructure for rapid VUS reclassification, and lowering of VUS rates will take time and resources. However, there is an urgent need for government, healthcare providers and researchers to invest time and effort immediately because managing VUS results is already having a direct impact on patient management and care, and as more genes are analyzed, the issues will only intensify.

## Additional file

Additional file 1: Table S1. BRCA1/2 and related cancer panel testing in the United States after the Supreme Court ruling in Myriad.

## Abbreviations

ACMG: American College of Medical Genetics
BIC: Breast Cancer Information Core
CPA: Clinical Pathology Accreditation
EHRs: Electronic health records
ELSI: Ethical, legal, and social implication
IARC: International Agency for Research on Cancer
ISO: International Organization for Standardization
NCBI: National Center for Biotechnology Information
NHGRI: National Human Genome Research Institute
PCORI: Patient-Centered Outcomes Research Institute
VUS: Variant of uncertain (or unknown) significance
WES: Whole-exome sequencing
WGS: Whole-genome sequencing

## References

[1] Lindor NM, Guidugli L, Wang X, Vallée MP, Monteiro ANA, Tavtigian S, Goldgar DE, Couch FJ (2012). A review of a multifactorial probability-based model for classification of BRCA1 and BRCA2 variants of uncertain significance (VUS). Hum Mutat.

[2] Eggington JM, Bowles K, Moyes K, Manley S, Esterling L, Sizemore S, Rosenthal E, Theisen A, Saam J, Arnell C (2014). A comprehensive laboratory‐based program for classification of variants of uncertain significance in hereditary cancer genes. Clin Genet.

[3] Dewey FE, Grove ME, Pan C, Goldstein BA, Bernstein JA, Chaib H, Merker JD, Goldfeder RL, Enns GM, David SP (2014). Clinical interpretation and implications of whole-genome sequencing. JAMA.

[4] Kurian AW, Hare EE, Mills MA, Kingham KE, McPherson L, Whittemore AS, McGuire V, Ladabaum U, Kobayashi Y, Lincoln SE (2014). Clinical evaluation of a multiple-gene sequencing panel for hereditary cancer risk assessment. J Clin Oncol.

[5] Futreal PA, Wooster RF, Ashworth A, Stratton MR. **Materials and methods relating to the identification and sequencing of the*****BRCA2*****cancer susceptibility gene and uses thereof.** *UK Patent GB 2307477 A. Filed 25 November, 1996; issued 29 May, 1997.*

[6] Miki Y, Swensen J, Shattuck-Eidens D, Futreal PA, Harshman K, Tavtigian S, Liu Q, Cochran C, Bennett LM, Ding W (1994). A strong candidate for the breast and ovarian cancer susceptibility gene *BRCA1*. Science.

[7] Skolnick MH, Goldgar DE, Miki Y, Swenson J, Kamb A, Harshman KD, Shattuck-Eidens DM, Tavtigian SV, Wiseman RW, Futreal PA. **17Q-linked breast and ovarian cancer susceptibility gene.** *US patent 5,747,282. Filed 7 June, 1995; issued 5 May, 1998.*

[8] Wooster R, Bignell G, Lancaster J, Swift S, Seal S, Mangion J, Collins N, Gregory S, Gumbs C, Micklem G (1995). Identification of the breast cancer susceptibility gene *BRCA2*. Nature.

[9] Howlader N, Noone A, Krapcho M, Garshell J, Miller D, Altekruse S, Kosary C, Yu M, Ruhl J, Tatalovich Z (2014). SEER Cancer Statistics Review, 1975–2011.

[10] Domchek SM, Weber BL (2006). Clinical management of *BRCA1* and *BRCA2* mutation carriers. Oncogene.

[11] Culver JO, Brinkerhoff CD, Clague J, Yang K, Singh KE, Sand SR, Weitzel JN (2013). Variants of uncertain significance in *BRCA* testing: evaluation of surgical decisions, risk perception, and cancer distress. Clin Genet.

[12] **Clinical Pathology Accreditation & Transfer of Accreditation to UKAS.** [http://www.ukas.com/services/CPA/Clinical_Pathology_Accreditation_CPA.asp]

[13] Frank TS, Deffenbaugh AM, Reid JE, Hulick M, Ward BE, Lingenfelter B, Gumpper KL, Scholl T, Tavtigian SV, Pruss DR (2002). Clinical characteristics of individuals with germline mutations in *BRCA1* and *BRCA2*: analysis of 10,000 individuals. J Clin Oncol.

[14] Plon SE, Eccles DM, Easton D, Foulkes WD, Genuardi M, Greenblatt MS, Hogervorst FBL, Hoogerbrugge N, Spurdle AB, Tavtigian SV (2008). Sequence variant classification and reporting: recommendations for improving the interpretation of cancer susceptibility genetic test results. Hum Mutat.

[15] Spurdle AB, Healey S, Devereau A, Hogervorst FB, Monteiro AN, Nathanson KL, Radice P, Stoppa-Lyonnet D, Tavtigian S, Wappenschmidt B (2012). ENIGMA–evidence-based network for the interpretation of germline mutant alleles: an international initiative to evaluate risk and clinical significance associated with sequence variation in BRCA1 and BRCA2 genes. Hum Mutat.

[16] **Breast Cancer Information Core: An Open Access On-Line Breast Cancer Mutation Data Base.** [http://research.nhgri.nih.gov/bic/]

[17] Lerman C, Narod S, Schulman K, Hughes C, Gomez-Caminero A, Bonney G, Gold K, Trock B, Main D, Lynch J, Fulmore C, Snyder C, Lemon SJ, Conway T, Tonin P, Lenoir G, Lynch H (1996). BRCA1 testing in families with hereditary breast-ovarian cancer: a prospective study of patient decision making and outcomes. JAMA.

[18] Tavtigian SV, Greenblatt MS, Goldgar DE, Boffetta P (2008). Assessing pathogenicity: overview of results from the IARC Unclassified Genetic Variants Working Group. Hum Mutat.

[19] Supreme Court of the United States (2013). Association for Molecular Pathology v. Myriad Genetics. Volume 133.

[20] Cook-Deegan R, Niehaus A (2014). After Myriad: genetic testing in the wake of recent supreme court decisions about gene patents. Curr Genet Med Rep.

[21] **Ambry Genetics: BRCA and Beyond.** [http://brcaandbeyond.com/]

[22] Lindor NM, Goldgar DE, Tavtigian SV, Plon SE, Couch FJ (2013). *BRCA1*/*2* sequence variants of uncertain significance: a primer for providers to assist in discussions and in medical management. Oncologist.

[23] US District Court for Utah (2014). Memorandum Decision and Order Denying Plaintiffs’ Motion for Preliminary Injunction.

[24] Baldwin AL, Cook-Deegan R (2013). Constructing narratives of heroism and villainy: Case study of Myriad’s BRACAnalysis® compared to Genentech’s Herceptin®. Genome Med.

[25] Wallis Y, Payne S, McAnulty C, Bodmer D, Sistermans E, Robertson K, Moore D, Abbs S, Deans Z, Devereau A (2013). Practice Guidelines for the Evaluation of Pathogenicity and the Reporting of Sequence Variants in Clinical Molecular Genetics.

[26] Richards CS, Bale S, Bellissimo DB, Das S, Grody WW, Hegde MR, Lyon E, Ward BE, Committee MSotALQA (2008). ACMG recommendations for standards for interpretation and reporting of sequence variations: revisions 2007. Genet Med.

[27] Domchek S, Weber BL (2008). Genetic variants of uncertain significance: flies in the ointment. J Clin Oncol.

[28] Miller-Samuel S, MacDonald DJ, Weitzel JN, Santiago F, Martino MA, Namey T, Augustyn A, Mueller R, Forman A, Bradbury AR (2011). Variants of uncertain significance in breast cancer-related genes: real-world implications for a clinical conundrum. Part one: clinical genetics recommendations. Semin Oncol.

[29] DeMichele A, Weber BL (2002). Risk management in *BRCA1* and *BRCA2* mutation carriers: lessons learned, challenges posed. J Clin Oncol.

[30] Hakem R, de la Pompa JL, Sirard C, Mo R, Woo M, Hakem A, Wakeham A, Potter J, Reitmair A, Billia F, Firpo E, Hui CC, Roberts J, Rossant J, Mak TW (1996). The tumor suppressor gene *Brca1* is required for embryonic cellular proliferation in the mouse. Cell.

[31] Radice P, De Summa S, Caleca L, Tommasi S (2011). Unclassified variants in *BRCA* genes: guidelines for interpretation. Ann Oncol.

[32] Pharoah PD, Dunning AM, Ponder BA, Easton DF (2004). Association studies for finding cancer-susceptibility genetic variants. Nat Rev Cancer.

[33] Easton Douglas F, Deffenbaugh Amie M, Pruss D, Frye C, Wenstrup Richard J, Allen-Brady K, Tavtigian Sean V, Monteiro Alvaro NA, Iversen Edwin S, Couch Fergus J, Goldgar David E (2007). A systematic genetic assessment of 1,433 sequence variants of unknown clinical significance in the *BRCA1* and *BRCA2* breast cancer–predisposition genes. Am J Hum Genet.

[34] American College of Medical Genetics and Genomics (2007). ACMG Standards and Guidelines for Clinical Genetics Laboratories.

[35] Claustres M, Kozich V, Dequeker E, Fowler B, Hehir-Kwa JY, Miller K, Oosterwijk C, Peterlin B, van Ravenswaaij-Arts C, Zimmermann U, Zuffardi O, Hastings RJ, Barton DE (2014). Recommendations for reporting results of diagnostic genetic testing (biochemical, cytogenetic and molecular genetic). Eur J Hum Genet.

[36] Feero W (2014). Clinical application of whole-genome sequencing: proceed with care. JAMA.

[37] **Myriad Genetics: All Products.** [http://www.myriad.com/products-services/all-products/overview/]

[38] **Ambry Genetics: Panels.** [http://www.ambrygen.com/panels]

[39] **Tests offered by GeneDx.** [https://www.genedx.com/test-catalog/available-tests/]

[40] **Invitae Genetics: Test Catalog.** [https://www.invitae.com/en/test-catalog/]

[41] **Quest Diagnostics: Test Center.** [http://www.questdiagnostics.com/testcenter/TestCenterHome.action]

[42] **LabCorp: Integrated Oncology, IntelliGEN.** [https://www.labcorp.com/.EdosPortlet/TestMenuLibrary?libName=File+Library&compName=L12684]

[43] **Counsyl: Family Prep Screen Clinical Information.** [https://www.counsyl.com/services/family-prep-screen/clinical-info/]

[44] **University of Washington, Department of Laboratory Medicine: Genetics and Solid Tumor Diagnostic Testing.** [http://depts.washington.edu/labweb/Divisions/MolDiag/MolDiagGen/index.htm]

[45] **UCLA Clinical Laboratory and Pathology Services: Lung Cancer Mutation Panel.** [http://www.crlonline.com/lco/action/doc/retrieve/docid/ucla/4688743]

[46] **The University of Chicago Genetic Services: Exome Sequencing.** [http://dnatesting.uchicago.edu/tests/670]

[47] **Emory Genetics Laboratory: Molecular Genetic Tests.** [http://geneticslab.emory.edu/tests/test-menu.php?filter=2]

[48] Domchek SM, Bradbury A, Garber JE, Offit K, Robson ME (2013). Multiplex genetic testing for cancer susceptibility: out on the high wire without a net?. J Clin Oncol.

[49] Biesecker LG, Green RC (2014). Diagnostic clinical genome and exome sequencing. N Engl J Med.

[50] Yang Y, Muzny DM, Xia F, Niu Z, Person R, Ding Y, Ward P, Braxton A, Wang M, Buhay C, Veeraraghavan N, Hawes A, Chiang T, Leduc M, Beuten J, Zhang J, He W, Scull J, Willis A, Landsverk M, Craigen WJ, Bekheirnia MR, Stray-Pedersen A, Liu P, Wen S, Alcaraz W, Cui H, Walkiewicz M, Reid J, Bainbridge M (2014). Molecular findings among patients referred for clinical whole-exome sequencing. JAMA.

[51] Ferrone CR, Levine DA, Tang LH, Allen PJ, Jarnagin W, Brennan MF, Offit K, Robson ME (2009). *BRCA* germline mutations in Jewish patients with pancreatic adenocarcinoma. J Clin Oncol.

[52] Finch A, Beiner M, Lubinski J, Lynch HT, Moller P, Rosen B, Murphy J, Ghadirian P, Friedman E, Foulkes WD, Kim-Sing C, Wagner T, Tung N, Couch F, Stoppa-Lyonnet D, Ainsworth P, Daly M, Pasini B, Gershoni-Baruch R, Eng C, Olopade OI, McLennan J, Karlan B, Weitzel J, Sun P, Narod SA, Hereditary Ovarian Cancer Clinical Study Group (2006). Salpingo-oophorectomy and the risk of ovarian, fallopian tube, and peritoneal cancers in women with a *BRCA1* or *BRCA2* mutation. JAMA.

[53] Caulfield T, Evans J, McGuire A, McCabe C, Bubela T, Cook-Deegan R, Fishman J, Hogarth S, Miller FA, Ravitsky V, Biesecker B, Borry P, Cho MK, Carroll JC, Etchegary H, Joly Y, Kato K, Lee SS, Rothenberg K, Sankar P, Szego MJ, Ossorio P, Pullman D, Rousseau F, Ungar WJ, Wilson B (2013). Reflections on the cost of “low-cost” whole genome sequencing: framing the health policy debate. PLoS Biol.

[54] Yang Y, Muzny DM, Reid JG, Bainbridge MN, Willis A, Ward PA, Braxton A, Beuten J, Xia F, Niu Z, Hardison M, Person R, Bekheirnia MR, Leduc MS, Kirby A, Pham P, Scull J, Wang M, Ding Y, Plon SE, Lupski JR, Beaudet AL, Gibbs RA, Eng CM (2013). Clinical whole-exome sequencing for the diagnosis of Mendelian disorders. N Engl J Med.

[55] Petrucelli N, Lazebnik N, Huelsman KM, Lazebnik RS (2002). Clinical interpretation and recommendations for patients with a variant of uncertain significance in *BRCA1* or *BRCA2*: a survey of genetic counseling practice. Genet Test.

[56] Richter S, Haroun I, Graham TC, Eisen A, Kiss A, Warner E (2013). Variants of unknown significance in BRCA testing: impact on risk perception, worry, prevention and counseling. Ann Oncol.

[57] Plon SE, Cooper HP, Parks B, Dhar SU, Kelly PA, Weinberg AD, Staggs S, Wang T, Hilsenbeck S (2011). Genetic testing and cancer risk management recommendations by physicians for at-risk relatives. Genet Med.

[58] Pyeritz RE (2011). The coming explosion in genetic testing–is there a duty to recontact. N Engl J Med.

[59] **Myriad Presents Clinical Data on Myriad myRisk™ Hereditary Cancer Test at ASCO.** [http://globenewswire.com/news-release/2014/06/02/640970/10083950/en/Myriad-Presents-Clinical-Data-on-Myriad-myRisk-TM-Hereditary-Cancer-Test-at-ASCO.html]

[60] **Myriad Genetics Announces Participation In Innovative Collaboration to Accelerate Hereditary Cancer Research.** [http://investor.myriad.com/releasedetail.cfm?releaseid=851822]

[61] Allyse M, Sayres L, Havard M, King J, Greely H, Hudgins L, Taylor J, Norton M, Cho M, Magnus D (2013). Best ethical practices for clinicians and laboratories in the provision of noninvasive prenatal testing. Prenat Diagn.

[62] Angrist M, Jamal L. **Living laboratory: whole-genome sequencing as a learning healthcare enterprise.** *Clin Genet.* 2014. doi:10.1111/cge.12461. 10.1111/cge.12461 PMC4302048 25045831

[63] Wilfond BS, Nolan K (1993). National policy development for the clinical application of genetic diagnostic technologies: lessons from cystic fibrosis. JAMA.

[64] Stanley CM, Sunyaev SR, Greenblatt MS, Oetting WS (2014). Clinically relevant variants–identifying, collecting, interpreting, and disseminating: the 2013 annual scientific meeting of the human genome variation society. Hum Mutat.

[65] **What is ClinVar?.** [http://www.ncbi.nlm.nih.gov/clinvar/intro/]

[66] **The Global Alliance for Genomics & Health.** [http://genomicsandhealth.org/] 10.1089/gtmb.2014.1555 24896853

[67] **Sharing Clinical Reports Project (SCRP).** [http://sharingclinicalreports.org/]

[68] **Free the Data.** [http://www.free-the-data.org/]

[69] **Multiple Institutions, Labs Collaborating on Registry to Study Rare Hereditary Cancer Genes.** [https://www.genomeweb.com/clinical-genomics/multiple-institutions-labs-collaborating-registry-study-rare-hereditary-cancer-g]

[70] Green RC, Berg JS, Grody WW, Kalia SS, Korf BR, Martin CL, McGuire AL, Nussbaum RL, O’Daniel JM, Ormond KE (2013). ACMG recommendations for reporting of incidental findings in clinical exome and genome sequencing. Genet Med.

[71] Parthasarathy S (2012). Building Genetic Medicine: Breast Cancer, Technology, and the Comparative Politics of Health Care.

[72] Gold ER, Carbone J (2010). Myriad Genetics: In the eye of the policy storm. Genet Med.

[73] **How a Gene-Patent Test Case Will Help Both Patients and Inventors.** [http://www.theglobeandmail.com/globe-debate/how-a-gene-patent-test-case-will-help-both-patients-and-inventors/article21437174/]

[74] van Zimmeren E, Nicol D, Gold R, Carbone J, Chandrasekharan S, Baldwin AL, Cook-Deegan R: **The BRCA patent controversies.** In *Breast Cancer Gene Research and Medical Practices: Transnational Perspectives in the Time of BRCA.* Edited by Gibbon S, Joseph G, Mozersky J, Nieden AZ, Palfner S. New York: Routledge; 2014:151–174.

[75] Nicol D, Nielsen JL, Liddicoat JE, Critchley C, Whitton T (2014). The special case of genetic testing. The Innovation Pool in Biotechnology: The Role of Patents in Facilitating Innovation.

[76] **ISO 15189:2012 Medical Laboratories -- Requirements for Quality and Competence.** [http://www.iso.org/iso/catalogue_detail?csnumber=56115]

[77] **Accreditation and Laboratory Improvement.** College of American Pathologists 2014 [http://www.cap.org/apps/cap.portal?_nfpb=true&_pageLabel=accreditation]

[78] DesRoches CM, Charles D, Furukawa MF, Joshi MS, Kralovec P, Mostashari F, Worzala C, Jha AK (2013). Adoption of electronic health records grows rapidly, but fewer than half of US hospitals had at least a basic system in 2012. Health Aff.

[79] Jha AK, DesRoches CM, Campbell EG, Donelan K, Rao SR, Ferris TG, Shields A, Rosenbaum S, Blumenthal D (2009). Use of electronic health records in U.S. hospitals. N Engl J Med.

[80] Jha AK, DesRoches CM, Kralovec PD, Joshi MS (2010). A progress report on electronic health records in U.S. hospitals. Health Aff.

[81] **PCORnet: The National Patient-Centered Clinical Research Network.** [http://www.pcori.org/funding-opportunities/pcornet-national-patient-centered-clinical-research-network/]

[82] **Genetic Alliance.** [http://www.geneticalliance.org/about]

[83] **Facing Our Risk of Cancer Empowered.** [http://www.facingourrisk.org/index.php]

[84] **DuchenneConnect.** [https://www.duchenneconnect.org/]

