# Supplementary material for: Variants of uncertain significance in BRCA: a harbinger of ethical and policy issues to come?
Source: Genome Med. 2014 Dec 19;6(12):121. doi: 10.1186/s13073-014-0121-3 (PMC4295298; doi:10.1186/s13073-014-0121-3)
Supplement: Additional file 1: Table S1. — BRCA1/2 and related cancer panel testing in the United States after the Supreme Court ruling in Myriad. [file 13073_2014_121_MOESM1_ESM.pdf]

Supplementary Table 1. BRCA1/2 and related cancer panel testing in the United States after the Supreme Court ruling in Myriad.

Gene panels that include more than BRCA 1/2 are highlighted in BLUE.

Company	Test	Test Description	Genes Analyzed	Method & Other Notes
**Myriad Genetics** 1996	Integrated/ Comprehensive BRACAnalysis®	Complete *BRCA1/2* sequence; 5 common large rearrangements	*BRCA1 & BRCA2*	**Methods:** Multiplexed quantitative PCR and microarray-CGH analysis.
	BRACAnalysis® (not comprehensive)	Now recommending Comprehensive BRACAnalysis® for most patients	*BRCA1 & BRCA2*	
	BRCA1 or BRCA2 Single Site BRACAnalysis®	Known familial mutations in *BRCA1* or *BRCA2*	BRCA1 & BRCA2	
	Multisite BRACAnalysis®	3 Ashkenazi Jewish founder mutations	*BRCA1 & BRCA2*: c.68_69delAG [185delAG], c.5266dupC [5382insC], and c.5946delT [6174delT]	
	BRACAnalysis Large Rearrangement Test (BART)		BRCA1 & BRCA2	
	MyRisk®	25 genes for 8 hereditary cancers (breast, ovarian, gastric, colorectal, pancreatic, melanoma, prostate, and endometrial).	BRCA1/2, MLH1, MLH2, PMS2, EPCAM, APC, MUTYH, CDKN2A, PALB2, STX11, PTEN, TP53, CDH1, BMPR1A, SMAD4, ATM, BARD1, BRIP1, CDK4, CHEK2, NBN, RAD51C, RAD51D	
**UCLA Diagnostic Molecular Pathology Laboratory** May 2012	BRCA1 & 2 Ashkenazi Jewish Mutations	3 Ashkenazi Jewish founder mutations	BRCA1 & BRCA2: c.68_69delAG [185delAG], c.5266dupC [5382insC], and c.5946delT [6174delT]	**Methods:** Sanger Sequencing. **Note:** Send out other samples to Myriad; only offer the Ashkenazi panel.

Company	Test	Test Description	Genes Analyzed	Method & Other Notes
**Ambry Genetics**<br>June 13, 2014	*BRCA1 or BRCA2* site specific analysis	Known familial mutations in BRCA1 or BRCA2	*BRCA1 or BRCA2*	**Methods:** NGS of coding exons. Deletion/duplication analysis using the multiplex ligation-dependent probe amplification (MLPA). BRCAplus uses a custom targeted microarray to identify gross deletions or duplications. OvaNext and CancerNext use NGS or Sanger sequencing. All mutations and VUS' are confirmed through Sanger sequencing.
	BRCA Ashkenazi Jewish panel	3 Ashkenazi Jewish founder mutations	BRCA1 & BRCA2: c.68_69delAG [185delAG], c.5266dupC [5382insC], and c.5946delT [6174delT]	
	BRCA1/2 Deletion/ Duplication Only	Rearrangement analysis	BRCA1 & BRCA2	
	BRCA1/2 Sequence + Deletion/Duplication	Seq. and rearrangement analysis	BRCA1 & BRCA2	
	BRCA Ashkenazi Jewish 3-site mutation panel w/ reflex to *BRCA1/2* Analysis if negative	3 founder mutations. If test comes back negative, reflex to full sequencing of *BRCA1/2*.	BRCA1 & BRCA2: c.68_69delAG [185delAG], c.5266dupC [5382insC], and c.5946delT [6174delT]. REFLEX to sequence of BRCA1 & BRCA2	
	BRCA1/2 analysis w/ reflex to BRCAplus if negative	Full seq. of *BRCA1/2*. If negative, del/dup analysis of 4 other high-risk genes.	BRCA1/2 with REFLEX to BRCA1/2, CDH1, PTEN, STK11 and TP53 if negative	
	*BRCA*plus®	6-gene high risk panel; Sequence and Del/Dup Analysis	BRCA1/2, CDH1, PTEN, STK11 and TP53	
	BreastNext®	18 gene Del/Dup Analysis	ATM, BARD1, BRCA1, BRCA2, BRIP1, CDH1, CHEK2, MRE11A, MUTYH, NBN, NF1, PALB2, PTEN, RAD50, RAD51C, RAD51D, STK11 and TP53	
	OvaNext®	Full gene seq. and del/dup analysis of *23 genes* for breast, ovarian and/or uterine cancers. Specific mutation analysis available for known familial mutations.	ATM, BARD1, BRCA1, BRCA2, BRIP1, CDH1, CHEK2, EPCAM, MLH1, MRE11A, MSH2, MSH6, MUTYH, NBN, NF1, PALB2, PMS2, PTEN, RAD50, RAD51C, RAD51D, STK11, and TP53.	
	CancerNext-Expanded	Full gene seq. of 43 and dup/del analysis of 42 genes for breast, colon, ovarian, uterine and other cancers. Specific mutation analysis available for known familial mutations.	APC, ATM, BARD1, BRCA1, BRCA2, BRIP1, BMPR1A, CDH1, CDK4, CDKN2A, CHEK2, EPCAM, FH, FLCN, MAX, MET, MITF, MLH1, MRE11A, MSH2, MSH6, MUTYH, NBN, NF1, PALB2, PMS2, PTEN, RAD50, RAD51C, RAD51D, RET, SDHA, SDHAF2, SDHB, SDHC, SDHD, SMAD4, STK11, TMEM127, TP53, TSC1, TSC2, and VHL	

Company	Test	Test Description	Genes Analyzed	Method & Other Notes
**Ambry Genetics** (continued)	CancerNext®	Full gene seq. and dup/del analysis of *28 genes* for breast, colon, ovarian, uterine and other cancers. Specific mutation analysis available for known familial mutations.	APC, ATM, BARD1, BRCA1, BRCA2, BRIP1, BMPR1A, CDH1, CDK4, CDKN2A, CHEK2, EPCAM, MLH1, MRE11A, MSH2, MSH6, MUTYH, NBN, NF1, PALB2, PMS2, PTEN, RAD50, RAD51C, RAD51D, SMAD4, STK11, and TP53	
**University of Washington** June 14, 2014	Single Gene Analysis	Sequencing for any gene, such as *BRCA1* or *BRCA2*	BRCA1 or BRCA2	**Methods:** NGS (Illumina HiSeq2000) **Note:** The clinical lab and the King lab are separate entities. The King Lab offers free BROCA testing for families who meet its testing criteria (only for subjects in King's genetic research studies). The clinical lab at UW provides commercial BROCA panel testing for patients who are referred by their providers.
	Known Familial Mutation	Point mutation analysis	BRCA1 or BRCA2	
	BRCA1/2 Ashkenazi Jewish 3-site	Test not performed by Washington; Samples are sent off to Mayo for testing and analysis	BRCA1 & BRCA2: c.68_69delAG [185delAG], c.5266dupC [5382insC], and c.5946delT [6174delT]	
	BRCA1/2 Complete Analysis	Sequencing and dup/del	BRCA1 & BRCA2	
	BROCA - Cancer Risk Panel	Complete sequence of genes and detection of large deletions, duplications, and mosaicism. Genes for breast or ovarian cancer, and also colorectal, endometrial, pancreatic, endocrine, or melanoma.	AKT1, APC, ATM, ATR, BAP1, BARD1, BMPR1A, BRCA1, BRCA2, BRIP1, CDH1, CDK4, CDKN2A, CHEK1, CHEK2, CTNNA1, FAM175A (Abraxas), GALNT12, GEN1, GREM1, HOXB13, MEN1, MLH1, MRE11A, MSH2 (+EPCAM), MSH6, MUTYH, NBN, PALB2, PIK3CA, PPM1D, PMS2, POLD1, POLE, PRSS1, PTEN, RAD50, RAD51, RAD51C, RAD51D, RET, SDHB, SDHC, SDHD, SMAD4, STK11, TP53, TP53BP1, VHL, and XRCC2	

Company	Test	Test Description	Genes Analyzed	Method & Other Notes
**Fulgent Therapeutics, LLC** mid-June 2013	Breast Ovarian Cancer NGS Panel	39 genes. Sequencing of 39 genes involved in hereditary breast and ovarian cancer predisposition	APC, ATM, ATR, AXIN2, BAP1, BARD1, BLM, BMPR1A, BRCA1, BRCA2, BRIP1, CDH1, CDK4, CDKN2A, CHEK2, CTNNB1, EPCAM, FANCC, HOXB13, MLH1, MRE11A, MSH2, MSH6, MUTYH, NBN, PALB2, PALLD, PMS2, PTEN, RAD50, RAD51, RAD51C, RAD51D, SMAD4, STK11, TP53, VHL, XRCC2, XRCC3	**Methods:** NGS (Illumina MiSeq®) for larger panels and now for BRCA1/2 analysis as well. Confirm with Sanger sequencing, sometimes use Ion Proton®. MLPA for del/dup. **Note:** If a VUS result is given, the lab will offer free of charge sequencing for any additional family members.
	BRCA1 and BRCA2 deletion/duplication analysis		BRCA1 & BRCA2	
	BRCA1 and BRCA2 full gene sequence analysis		BRCA1 & BRCA2	
	Hereditary Cancer Panel (HCP)	Seq. (no rearrangement analysis) of all exonic and proximal intronic sequences of 112 genes involved in hereditary cancer predisposition (breast, colorectal, and cancers of the ovary, kidney, bladder, liver, stomach, gall bladder, prostate, skin, pancreas, brain and others).	APC,AIP, ATM, ATR, AXIN2, BAP1, BARD1, BLM, BMPR1A, BRCA1, BRCA2, BRIP1, BUB1B, CDH1, CDK4, CDKN1B , CDKN2A, CHEK2, CTNNB1, CYLD, DDB2, DICER1, EGFR, EGLN1, EPCAM, ERCC2, ERCC3, ERCC4, ERCC5, EXO1, EXT1, EXT2, FANCA, FANCB, FANCC, FANCD2, FANCE, FANCF, FANCG, FANCI, FANCL, FANCM, FH, FLCN, GALNT12, GPC3, HOXB13, HRAS, KIF1B, KIT, MAX, MC1R, MEN1, MET, MITF, MLH1, MPL, MRE11A, MSH2, MSH3, MSH6, MUTYH, NBN, NF1, NF2, PALB2, PDGFRA, PICALM, PMS1, PMS2, POLD1, PRKAR1A, PRKDC, PRSS1, PTCH1, PTEN, PTPN11, RAD50, RAD51, RAD51C, RAD51D, RB1, RBBP8, RBM15, RECQL4, RET, ROBO2, SBDS, SDHA, SDHAF2, SDHB, SDHC, SDHD, SLX4, SMAD4, SMARCB1, STK11, SUFU, TERT, TMEM127, TP53, TSC1, TSC2, TSHR, TYR, VHL, WRN, WT1, XPA, XPC, XRCC2, XRCC3	

Company	Test	Test Description	Genes Analyzed	Method & Other Notes
**GeneDx**<br>Gaithersburg, MD<br>(Subsidiary of **Bio-Reference Laboratories, Inc**)<br>August 28, 2013	BRCA1/2 Sequencing		BRCA1 & BRCA2	**Method:** Capillary sequencing and Exon ArrayCGH. Cancer Panels use NGS (Illumina MiSeq®).<br>**Note:** GeneDx will offer to test additional family members for the VUS for free upon detailed review of the clinical and family history.
	BRCA1/2 Del/Dup		BRCA1 & BRCA2	
	BRCA1/2 Familial Variant	Checks for known point mutations	BRCA1 & BRCA2	
	BRCA1/2 Sequencing and Del/Dup Analysis		BRCA1 & BRCA2	
	BRCA1/2 Ashkenazi Founder Mutation Panel		BRCA1 & BRCA2: c.68_69delAG [185delAG], c.5266dupC [5382insC], and c.5946delT [6174delT]	
	OncoGeneDx Comprehensive Cancer Panel	Seq. and Del/Dup Analysis for 29 genes for: Attenuated Familial Adenomatous Polyposis (AFAP), Breast Cancer, Colorectal Cancer, Endometrial Cancer, Familial Adenomatous Polyposis (FAP), Ovarian Cancer, Pancreatic Cancer, and Uterine Cancer	APC, ATM, AXIN2, BARD1, BMPR1A, BRCA1, BRCA2, BRIP1, CDH1, CDK4, CDKN2A, CHEK2, EPCAM, FANCC, MLH1, MSH2, MSH6, MUTYH, NBN, PALB2, PMS2, PTEN, RAD51C, RAD51D, SMAD4, STK11, TP53, VHL, XRCC2	
	High/Moderate Risk Panel for Breast Cancer	Seq. Analysis and/or Exon-Level del/dup analysis of 20 genes for hereditary cancer	APC, ATM, BMPR1A, BRCA1, BRCA2, CDH1, CDKN2A, CHEK2, EPCAM, MLH1, MSH2, MSH6, MUTYH, PALB2, PMS2, PTEN, SMAD4, STK11, TP53, VH	
	Breast Cancer High Risk Panel	6 genes high risk panel	BRCA1, BRCA2, CHH1, PTEN, STK11, TP53	
	Breast/Ovarian Cancer Panel	Seq. and Del/Dup Analysis for 21 genes for breast and ovarian cancer	ATM, BARD1, BRCA1, BRCA2, BRIP1, CDH1, CHEK2, EPCAM, FANCC, MLH1, MSH2, MSH6, NBN, PALB2, PMS2, PTEN, RAD51C, RAD51D, STK11, TP53, XRCC2	

Company	Test	Test Description	Genes Analyzed	Method & Other Notes
**Quest Diagnostics** mid-October 2013	BRCAvantage® Comprehensive Evaluation	Detection of point mutations, deletions, duplications, and rearrangements in *BRCA1/2*	BRCA1 & BRCA2	**<u>Methods:</u>** NGS for sequencing and multiplex ligand-dependent probe amplification (MLPA) to detect deletions, duplications, and rearrangements.
	BRCAvantage® Ashkenazi Jewish Screen w/ reflex to Comprehensive Evaluation	If Ashkenazi Screen is negative, BRCAvantage, Comprehensive will be performed		
	BRCAvantage® Ashkenazi Jewish Evaluation	Detection of 3 founder mutations	BRCA1 & BRCA2: c.68_69delAG [185delAG], c.5266dupC [5382insC], and c.5946delT [6174delT]	
	BRCAvantage® Single Site	Detection of a known familial mutation in *BRCA1/2*.	BRCA1 or BRCA2	
	BRCAvantage® Rearrangement Evaluation	Detection of deletions, duplications, and rearrangements in *BRCA1/2*.	BRCA1 & BRCA2	

Company	Test	Test Description	Genes Analyzed	Method & Other Notes
**The University of Chicago Genetic Services** November 20, 2013	BRCA1 and BRCA2 familial testing	Testing for known familial BRCA1/2 mutations just by sequence analysis.	*BRCA1 & BRCA2*	**<u>Methods:</u>** Sanger Sequencing. PCR for dup/del.
	Custom mutation sequencing	Seq. of any previously identified gene/ Test costs $390 for additional family members.		
	Custom del/dup testing	Custom deletion/duplication testing by quantitative PCR. Test costs $450 for additional family members.	dependent	
	BRCA1 and BRCA2 founder mutations		BRCA1 & BRCA2: c.68_69delAG [185delAG], c.5266dupC [5382insC], and c.5946delT [6174delT]	
Laboratory Corporation of America Holdings **(LabCorp)** December 2, 2013	BRCA1 Targeted Analysis	Just sequencing	*BRCA1*	**<u>Methods:</u>** Sanger sequencing for sequencing. Multiplex ligation-dependent probe amplification (MLPA) platform for del/dup.
	BRCA2 Targeted Analysis	Just sequencing	BRCA2	
	BRCA1/2 Comprehensive Analysis (BRCAssure®)	Sequencing and del/dup analysis.	*BRCA1/2*	
	BRCA1/2 Deletion/Duplication Analysis (BRCAssure®)	Just del/dup analysis.	*BRCA1/2*	
	BRCA1/2 Ashkenazi Jewish Profile (BRCAssure®)	Just sequencing, not del/dup or rearrangements	BRCA1 & BRCA2: c.68_69delAG [185delAG], c.5266dupC [5382insC], and c.5946delT [6174delT]	

Company	Test	Test Description	Genes Analyzed	Method & Other Notes
**Invitae**<br>December 2013	Hereditary breast and ovarian cancer syndrome	Seq. and dup/del analysis for hereditary breast and ovarian cancer	BRCA1 & BRCA2	**Methods:** NGS **Note:** Invitae offers any test and every test for one price of $1,500. In addition to choosing from among their various multi-gene panels, you can design your own test.
	High-Risk Hereditary Breast Cancers	Seq. and dup/del analysis of 7 genes for: Hereditary breast and ovarian cancer syndrome, PTEN hamartoma tumor syndrome (Cowden syndrome), Li-Fraumeni syndrome, Peutz-Jeghers syndrome, Hereditary diffuse gastric cancer	BRCA1, BRCA2, PALB2, PTEN, TP53, STK11, CDH1	
	Women's Hereditary Cancers	Seq. and dup/del analysis of 17 genes for Hereditary Breast, Ovarian, and Endometrial Cancer Syndromes	BRCA1, BRCA2, PTEN, TP53, MLH1, MSH2, MSH6, EPCAM, PMS2, STK11, CDH1, CHEK2, RAD51C, BRIP1, PALB2, NBN, ATM	
	Hereditary Cancer Syndromes	Seq. and dup/del analysis for 29 genes for: Hereditary breast and ovarian cancer syndrome, PTEN hamartoma tumor syndrome, Li-Fraumeni syndrome, Lynch syndrome, Familial adenomatous polyposis, Juvenile polyposis syndrome, Peutz-Jeghers syndrome, MYH-associated polyposis syndrome, Hereditary diffuse gastric cancer, Familial cutaneous melanoma, Familial pancreatic adenocarcinoma, Hereditary papillary renal cell carcinoma, Multiple endocrine neoplasia, type 1, Multiple endocrine neoplasia, type 2, Basal cell nevus syndrome, Von Hippel-Lindau syndrome, Moderate-risk breast cancer susceptibility	BRCA1, BRCA2, PTEN, TP53, MLH1, MSH2, MSH6, EPCAM, PMS2, APC, BMPR1A, SMAD4, STK11, MUTYH, CDH1, CDK4, CDKN2A, PALLD, MET, MEN1, RET, PTCH1, VHL, CHEK2, BRIP1, PALB2, RAD51C, NBN, ATM	
	Family Testing Services	Targeted mutation analysis offered to family members of those who have received a positive result on one of the genetic tests.	dependent	

Company	Test	Test Description	Genes Analyzed	Method & Other Notes
**Center for Human Genetics, Inc** January 2014	3 founder mutations		BRCA1 & BRCA2: c.68_69delAG [185delAG], c.5266dupC [5382insC], and c.5946delT [6174delT]	**Methods:** Targeted variant analysis (PCR with RFLP). Have "plans to offer full-gene seq. and dup/del analysis in the future."
**Counsyl** Spring 2014	Counsyl Inherited Cancer (BRCA) Screen	Sequence and dup/del analysis	BRCA1 & BRCA2	**Method:** Next-gen Illumina-based assay, but custom build their own hardware and software **Note:** Anyone who takes a Counsyl screen can opt for a complimentary phone consultation.
**University of Michigan State Testing Lab** Spring 2014	Tier 1 BRCA Mutation Panel" BRCA1 & BRCA2 sequencing		BRCA1 & BRCA2	**Methods:** Bi-directional Sanger Sequence Analysis w/ Applied Biosystems 3730 capillary sequencing instrument. **Note:** Require a **$85 pathology interpretation**, so this cost must be added ***onto*** each of the test costs listed.
	Tier 2 BRCA Mutation Panel: BRCA1 & BRCA2 dup/del		BRCA1 & BRCA2	
	BRCA Ashkenazi Jewish Founder Mutations		BRCA1 & BRCA2: c.68_69delAG [185delAG], c.5266dupC [5382insC], and c.5946delT [6174delT]	
	BRCA1/2 Targeted Sequencing, Familial		BRCA1 or BRCA2	
	BRCA1/2 Gene Sequencing		BRCA1 & BRCA2	
	BRCA1/2 del/dup analysis		BRCA1 & BRCA2	

Company	Test	Test Description	Genes Analyzed	Method & Other Notes
**Pathway Genomics**<br>June 3, 2014	BRCATrue®	Sequencing and rearrangement analysis. Price includes full gene sequencing and common rearrangments, but unclear if there is an additional price for uncommon rearrangements.	BRCA1 & BRCA2	**Methods:** NGS and confirms any mutaitons with Sanger. "Large gene rearrangements (large deletions or duplications) within the BRCA1 and BRCA2 genes are detected using quantitative PCR (qPCR). Positive results are confirmed by array comparative genomic hybridization (aCGH)."<br>**Note:** Have a "One for One" program; for every one test ordered, one is donated to a person in need.
**BCM Medical Genetics Laboratories**<br>Spring 2014	BRCA1 Gene Sequencing by Massively Parallel Sequencing		BRCA1	**Methods:** NGS sequencing. Array CGH Analysis (aCGH), and Multiplex Ligation-dependent Probe Amplification (MLPA).<br>**Payment Notes**: Baylor has 2 price points, the lower prices correspond to the *Institutional* and *Self-pay* costs, while the higher prices are billed to *Insurance* companies.
	BRCA1 Sequence Analysis (Familial Mutation/Variant Analysis)		BRCA1	
	BRCA1 Sequence Analysis (Prenatal Sequence Analysis)		BRCA1	
	BRCA2 Gene Sequencing by Massively Parallel Sequencing		BRCA2	
	BRCA2 Sequence Analysis (Familial Mutation/Variant Analysis)		BRCA2	
	BRCA2 Sequence Analysis (Prenatal Sequence Analysis)		BRCA2	
	BRCA1/2 Sequence & Del/Dup Analysis	Test is sent to Counsyl, NOT done in Baylor's lab	BRCA1, BRCA2	

Company	Test	Test Description	Genes Analyzed	Method & Other Notes
**BCM Medical Genetics Laboratories** (continued)	Comprehensive Hereditary Cancer Panel	61 genes	ALK, APC, ATM, BARD1, BMPR1A, BRCA1, BRCA2, BRIP1, CBL, CDC73, CDH1, CDK4, CDKN1C, CDKN2A, CEBPA, CHEK2, ENG, EPCAM, FH, FLCN, GATA2, GPC3, MAX, MEN1, MET, MLH1, MRE11A, MSH2, MSH6, MUTYH, NBN, NF2, PALB2, PAX5, PHOX2B, PMS1, PMS2, PRF1, PRKAR1A, PTCH1, PTEN, PTPN11, RAD50, RAD51C, RAD51D, RET, RUNX1, SBDS, SDHA, SDHAF2, SDHB, SDHC, SDHD, SMAD4, STK11, SUFU, TMEM127, TP53, TSHR, VHL, and WT1	
	Hereditary Breast/Ovarian/ Endometrial Cancer Panel	23 genes	ATM, BARD1, BRCA1, BRCA2, BRIP1, CDH1, CHEK2, EPCAM, MLH1, MRE11A, MSH2, MSH6, MUTYH, NBN, PALB2, PMS1, PMS2, PTEN, RAD50, RAD51C, RAD51D, STK11, and TP53.	
	Hereditary High Risk Breast Cancer Panel	7 genes	BRCA1, BRCA2, CDH1, PALB2, PTEN, STK11, and TP53.	

Company	Test	Test Description	Genes Analyzed	Method & Other Notes
**Emory Genetics Lab** Spring 2014	BRCA1/BRCA2 Gene Seq. Panel		BRCA1, BRCA2	**Methods.** Sequencing by NGS. Sequence analysis is required before dup/del analysis (using targetted CGH array).
	BRCA1/BRCA2 Del/Dup Panel			
	BRCA1/BRCA2 Gene Seq. and Del/Dup Panel		BRCA1, BRCA2	
	High Risk Breast Cancer: Seq. and Del/dup Panel		BRCA1, BRCA2, CDH1, PALB2, PTEN, STK11, TP53	
	Breast and Ovarian Cancer: Seq. and Del/dup Panel		ATM, BRCA1, BRCA2, BRIP1, CDH1, CHEK2, EPCAM, MLH1, MSH2, MSH6, MUTYH, NBN, PALB2, PMS1, PMS2, PTEN, RAD51C, RAD51D, STK11, TP53	
	Hereditary Cancer Syndrome: Sequencing Panel		ALK, APC, ATM, BMPR1A, BRCA1, BRCA2, BRIP1, CDC73, CDH1, CDKN1C, CDKN2A, CHEK2, EPCAM, FH, FLCN, GPC3, MAX, MEN1, MET, MLH1, MSH2, MSH6, MUTYH, NBN, NF2, PALB2, PHOX2B, PMS1, PMS2, PRKAR1A, PTCH1, PTEN, RAD51C, RAD51D, RET, SDHAF2, SDHB, SDHC, SDHD, SMAD4, STK11, SUFU, TMEM127, TP53, VHL, WT1	

Presented in chronological order, with the first company to offer testing listed first (Myriad Genetics). This list is not intended to be exhaustive, and these companies may have changed their test offerings since the publication of this table. Many of these companies have released additional tests or panels, especially those that include more than *BRCA1/2*.

Adapted from: Cook-Deegan R, Niehaus A: **After Myriad: Genetic Testing in the Wake of Recent Supreme Court Decisions about Gene Patents.** *Curr Genet Med Rep* 2014, **2:**223-241.
